# Computational Intelligence and Metaheuristic Algorithms with Applications

**DOI:** 10.1155/2014/425853

**Published:** 2014-12-31

**Authors:** Xin-She Yang, Su Fong Chien, Tiew On Ting

**Affiliations:** ^1^School of Science and Technology, Middlesex University, London NW4 4BT, UK; ^2^Strategic Advanced Research (StAR), Mathematical Modeling Lab, MIMOS Berhad, Technology Park Malaysia, Kuala Lumpur, Malaysia; ^3^Department of Electrical and Electronic Engineering, Xi'an Jiaotong-Liverpool University, No. 111 Ren'ai Road, HET, SIP, Suzhou, Jiangsu 215123, China

## 1. Introduction

Nature-inspired metaheuristic algorithms have become powerful and popular in computational intelligence and many applications. There are some important developments in recent years, and this special issue aims to provide a timely review of such developments, including ant colony optimization, bat algorithm, cuckoo search, particle swarm optimization, genetic algorithms, support vector machine, neural networks, and others. In addition, these algorithms have been applied in a diverse range of applications, and some of these latest applications are also summarized here.

Computational intelligence and metaheuristic algorithms have become increasingly popular in computer science, artificial intelligence, machine learning, engineering design, data mining, image processing, and data-intensive applications. Most algorithms in computational intelligence and optimization are based on swarm intelligence (SI) [1, 2]. For example, both particle swarm optimization [1] and cuckoo search [3] have attracted much attention in science and engineering. They both can effectively deal with continuous problems [2] and combinatorial problems [4]. These algorithms are very different from the conventional evolutionary algorithms such as genetic algorithms and simulated annealing [5, 6] and other heuristics [7].

Many new optimization algorithms are based on the so-called swarm intelligence (SI) with diverse characteristics in mimicking natural systems [1, 2]. Consequently, different algorithms may have different features and thus may behave differently, even with different efficiencies. However, It still lacks in-depth understanding why these algorithms work well and exactly under what conditions, though there were some good studies that may provide insight into algorithms [2, 8].

This special issue focuses on the recent developments of SI-based metaheuristic algorithms and their diverse applications as well as theoretical studies. Therefore, this paper is organized as follows.  Section 2 provides an introduction and comparison of the so-called infinite monkey theorem and metaheuristics, followed by the brief review of computational intelligence and metaheuristics in Section 3. Then, Section 4 touches briefly the state-of-the-art developments, and finally, Section 5 provides some open problems about some key issues concerning computational intelligence and metaheuristics.

## 2. Monkeys, Shakespeare, and Metaheuristics

There is a well-known thought experiment, called the infinite monkey theorem, which states that the probability of producing any given text will almost surely be one if an infinite number of monkeys randomly type for an infinitely long time [9, 10]. In other words, the infinite monkeys can be expected to reproduce the whole works of Shakespeare. For example, to reproduce the text “swarm intelligence” (18 characters including the space), for a random typing sequence of *n* characters on a 101-key computer keyboard, the probability of a consecutive 18-character random string to be “swarm intelligence” is *p*_*s*_ = (1/101)^18^ ≈ 8.4 × 10^−37^, which is extremely small. However, the importance here is that this probability is not zero. Therefore, for an infinitely long sequence *n* → *∞*, the probability of reproducing the collected works of Shakespeare is one, though the formal rigorous mathematical analysis requires Borel-Cantelli lemma [9, 11].

Conversely, we can propose a finite monkey theorem without proof. For a given finite number of monkeys typing for a fixed amount of time, what is the probability of reproducing any piece of text such as this paper?

In many ways, heuristic and metaheuristic algorithms have some similarities to the infinite monkey approach. Monkeys type randomly and, ultimately, some meaningful high-quality text may appear. Similarly, most stochastic algorithms use randomization to increase the search capability. If such algorithms are executed for a sufficiently long time with multiple runs, it can be expected that the global optimality of a given problem can be reached or found. In theory, it may take infinitely long to guarantee such optimality, but, in practice, it can take many thousands or even millions of iterations. If we consider the optimality as an important piece of work of Shakespeare, the infinite monkeys should be able to reproduce or achieve it in an infinite amount of time.

However, there are some key differences between the heuristic algorithms and the infinite monkey approach. First, monkeys randomly type without any memory or learning processing, and each key input is independent of another. Heuristic algorithms try to learn from history and the past moves so as to generate new, better moves or solutions [7]. Second, random monkeys do not select what has been typed, while algorithms try to select the best solutions or the fittest solutions [5]. Third, monkeys use purely stochastic components, while all heuristic algorithms use both deterministic and stochastic components. Finally, monkey typing at most is equivalent to a random search on a flat landscape, while heuristic algorithms are often cleverly constructed to use the landscape information in combination with history (memory) and selection. All these differences ensure that heuristic algorithms are far better than the random monkey-typing approach.

In addition, metaheuristics are usually considered as a higher level of heuristics, because metaheuristic algorithms are not simple trial-and-error approaches and metaheuristics are designed to learn from past solutions, to be biased towards better moves, to select the best solutions, and to construct sophisticated search moves. Therefore, metaheuristics can be much better than heuristic algorithms and can definitely be far more efficient than random monkey-typing approaches.

## 3. Computational Intelligence and Metaheuristics

Computational intelligence has been in active development for many years. Classical methods and algorithms such as machine learning methods, classifications and cluster methods, and data mining techniques are all well established, though constant improvements and refinements are being carried out. For example, neural networks and support vector machines have been around for a few decades, and they have been applied to almost every area of science and engineering [12, 13]. However, it was mainly in the 1990s when these two methods became truly popular, when the mass computer facilities become affordable with the steady increase of the computational speed.

Nowadays computational intelligence has permeated into many applications directly or indirectly. Accompanying this expansion, nature-inspired metaheuristic algorithms begin to demonstrate promising power in computational intelligence and many other areas [14]. For example, cuckoo search has been used in optimizing truss structures [15] and other applications [3], while a hybrid approach combining a two-stage eagle strategy with differential evolution can save computational efforts [16]. New algorithms emerge almost every year with a trend of speedup.

Algorithms which appeared in the last five years include bat algorithm [17], cuckoo search [3], flower pollination algorithm [18], and others, which are in addition to the popular and well-accepted algorithms such as particle swarm optimization, ant colony optimization, firefly algorithm, differential evolution, and genetic algorithms. Different algorithms have different sources of inspiration, and they can also perform differently [1, 2]. For example, among most recent, bioinspired algorithms, flower pollination algorithm (FPA), or flower algorithm (FA) for simplicity, was developed by Xin-She Yang, which has demonstrated very good efficiency in solving both single optimization and multiobjective optimization problems [18]. Both the flower algorithm and the cuckoo search use more subtle Lévy flights instead of standard Gaussian random walks [19].

However, some efficient approaches can be based on the combination of different algorithms, and the eagle strategy is a two-stage strategy combining a coarse explorative stage and an intensive exploitative stage in an iterative manner [16].

Applications can be very diverse, from structural optimization [15] to energy efficient telecommunications [20]. Detailed list of applications can be found in recent review articles [3, 17] or books [2].

## 4. State-of-the-Art Developments

As the developments are active and extensive, it is not possible to cover a good part of the recent advances in a single special issue. Therefore, this special issue can only provide a timely snapshot of the state-of-the-art developments. The responses to this special issue were overwhelming, and more than 100 submissions were received. After going through the rigorous peer-review process, 32 papers have been accepted for this issue. A brief summary of these papers is given below.

E. Cuevas et al. provide a study of multimodal optimization using the cuckoo search algorithm, while E. Saraç and S. A. Özel carry out web page classification using ant colony optimization and O. Elizarraras et al. obtain better performance in ad hoc network using genetic algorithms. In addition, K. Yang et al. provided a multiobjective memetic estimation based on incremental local search, and S. Darzi et al. solve a beam enhancement problem using particle swarm optimization and other approaches, followed by the study of routing in cognitive radio ad hoc networks by H. A. A. Al-Rawi et al. and the feature extraction of flotation froth images using a combined approach of shuffled cuckoo search and BP neural networks by J.-s. Wang et al. Furthermore, A. U. Ahmed et al. provide user categorization for closed access femtocell network and N. A. Ab Aziz et al. present a synchronous-asynchronous particle swarm optimization approach.

On the other hand, S. Lee and S. Shin carry out gait signal analysis using similarity measures, and J. Wang et al. use improved ant colony optimization for process planning, while S. Deng and A. Sakurai use multiple kernel learning approach in combination with differential evolution to model EUR/USD trading problems, followed by the optimization of virtual machine deployment by Y.-S. Dong et al. and fault detection of aircraft system by random forest algorithm and similarity measures by S. Lee et al. In addition, S. Kim presents an adaptive MANET multigraph routing approach based simulated annealing. Moreover, solution quality assessment in the context of swarm intelligence has been attempted by Z. Zhang et al. and application of model and algorithms in cognitive radio networks has been carried out by K.-L. A. Yau et al.

Further algorithm developments and enhancements include the study of the mean-variance portfolio optimization by using the firefly algorithm by N. Bacanin and M. Tuba, the global support curve data fitting via the cuckoo search with Lévy flights by A. Gálvez et al., the uncertain portfolio selection by artificial bee colony by W. Chen, and fuzzy partitioning problems by island grouping genetic algorithm approach by S. Salcedo-Sanz et al. In addition, Y. Zhou et al. present a cloud model based bat algorithm, while I. Fister Jr. et al. propose novel reasoning in the context of PSO using RDF and SPARQL, followed by J.-h. Yi et al.'s detailed study of back propagation optimization by the cuckoo search algorithm.

In addition to the above applications in networks, planning, and feature selection, more applications include the diagnosis of clinical diseases using PSO-based support vector machine with cuckoo search by X. Liu and H. Fu, phase equilibrium thermodynamic calculations using nature-inspired metaheuristic algorithms by S.-E. K. Fateen and A. Bonilla-Petriciolet, query workload optimization of cloud data warehouse by T. Dokeroglu et al., and crop-mix planning decision using multiobjective differential evolution by O. Adekanmbi et al.

S. Fong et al. propose ways to enhance performance of K-means clustering by using nature-inspired optimization algorithms, while A. Alihodzic and M. Tuba carry out multilevel image thresholding by using the improved bat algorithm. In addition, F. Gómez-Vela and N. Díaz-Díaz use gene-gene interaction for gene network biological validity, while D. Aguirre-Guerrero et al. provide a fair packet delivery method with congestion control in wireless sensor network, and N. Bouhmala solves MAX-SAT problems using a variable neighbourhood approach. In parallel with the above developments, T. O. Ting et al. tune Kalman filter parameters using genetic algorithms for battery management, and E. Osaba et al. present a golden ball algorithm for solving routing problems. Last but not least, C. Lagos et al. compare evolutionary strategies in the text of the biobjective cultural algorithm.

As we can see from the above extensive list of papers, the current studies concern a diverse range of real-world applications as well as algorithm developments and analysis.

## 5. Open Questions

In fact, there is still a significant gap between theory and practice. Most metaheuristic algorithms have successful applications in practice, but their mathematical analysis lags far behind. In fact, apart from a few limited results about the convergence and stability concerning particle swarm optimization, genetic algorithms, simulated annealing, and others [21, 22], many algorithms do not have theoretical analysis in the literature. Therefore, we may know that they can work well in practice, but we hardly understand why they work and how to improve them with a good understanding of their working mechanisms.

In addition, there is a well-known “no-free-lunch” theorem which concerns the average performance for solving all problems [23]. However, this theorem is only valid under strict conditions such as the assumptions of closed under permutation of solution sequences and nonrevising assumption of the search points. In fact, for coevolutionary approaches, there are potentially free lunches [24].

There are many key issues that need to be addressed in the context of computational intelligence and metaheuristic algorithms. To list all these problems may require a lengthy article to provide sufficient details for each key issue. However, we believe that the following open problems are worth emphasizing.

- It still lacks a general mathematical framework for analyzing the convergence and stability of metaheuristic algorithms. There are some good results using Markov chains, dynamic systems, and self-organization theory, but a systematic framework is yet to be developed.
- Parameter tuning is still a time-consuming process for tuning algorithms. How to best tune an algorithm so that it can work for a wide range of problems is still an unsolved problem. In fact, it is a hyperoptimization problem; that is, it is the optimization of an optimization algorithm.
- How can we solve high-dimensional problems effectively? At the moment, most case studies using metaheuristic algorithms are small-scale problems. It is not clear if these algorithms are scalable to deal with large-scale problems effectively.
- Discrete problems and combinatorial optimization, especially those NP-hard problems, are still very challenging to solve. Though studies indicate that metaheuristic algorithms can be effective alternatives [2], it is still at early stage of the research. More studies are highly needed.

Obviously, these challenges also pose great opportunities for researchers. It can be expected that any progress in the above areas will provide great insight into the understanding of metaheuristic algorithms and their capabilities in solving a diverse range of problems in real-world applications.

*Xin-She Yang* *Xin-She Yang* *Su Fong Chien* *Su Fong Chien* *Tiew On Ting* *Tiew On Ting*
